# Echocardiography and EuroSCORE II for the stratification of low-gradient severe aortic stenosis and preserved left ventricular ejection fraction

**DOI:** 10.1007/s10554-021-02373-2

**Published:** 2021-08-14

**Authors:** Yan Fan, Hong Shen, Brandon Stacey, David Zhao, Robert J. Applegate, Neal D. Kon, Edward H. Kincaid, Sanjay K. Gandhi, Min Pu

**Affiliations:** 1 Section on Cardiovascular Medicine, Wake Forest Baptist Medical Center, Medical Center Boulevard, Winston-Salem, NC 27157 USA; 2 Section of Cardiothoracic Surgery, Wake Forest Baptist Medical Center, Winston-Salem, USA; 3 First Hospital, Peking University, Beijing, China

**Keywords:** Aortic stenosis, EuroSCORE II, Echocardiography, Aortic valve intervention

## Abstract

The purpose of this study was to explore the utility of echocardiography and the EuroSCORE II in stratifying patients with low-gradient severe aortic stenosis (LG SAS) and preserved left ventricular ejection fraction (LVEF ≥ 50%) with or without aortic valve intervention (AVI). The study included 323 patients with LG SAS (aortic valve area ≤ 1.0 cm^2^ and mean pressure gradient < 40 mmHg). Patients were divided into two groups: a high-risk group (EuroSCORE II ≥ 4%, n = 115) and a low-risk group (EuroSCORE II < 4%, n = 208). Echocardiographic and clinical characteristics were analyzed. All-cause mortality was used as a clinical outcome during mean follow-up of 2 ± 1.3 years. Two-year cumulative survival was significantly lower in the high-risk group than the low-risk patients (62.3% vs. 81.7%, p = 0.001). AVI tended to reduce mortality in the high-risk patients (70% vs. 59%; p = 0.065). It did not significantly reduce mortality in the low-risk patients (82.8% with AVI vs. 81.2%, p = 0.68). Multivariable analysis identified heart failure, renal dysfunction and stroke volume index (SVi) as independent predictors for mortality. The study suggested that individualization of AVI based on risk stratification could be considered in a patient with LG SAS and preserved LVEF.

## Introduction

**Fig. 1:**
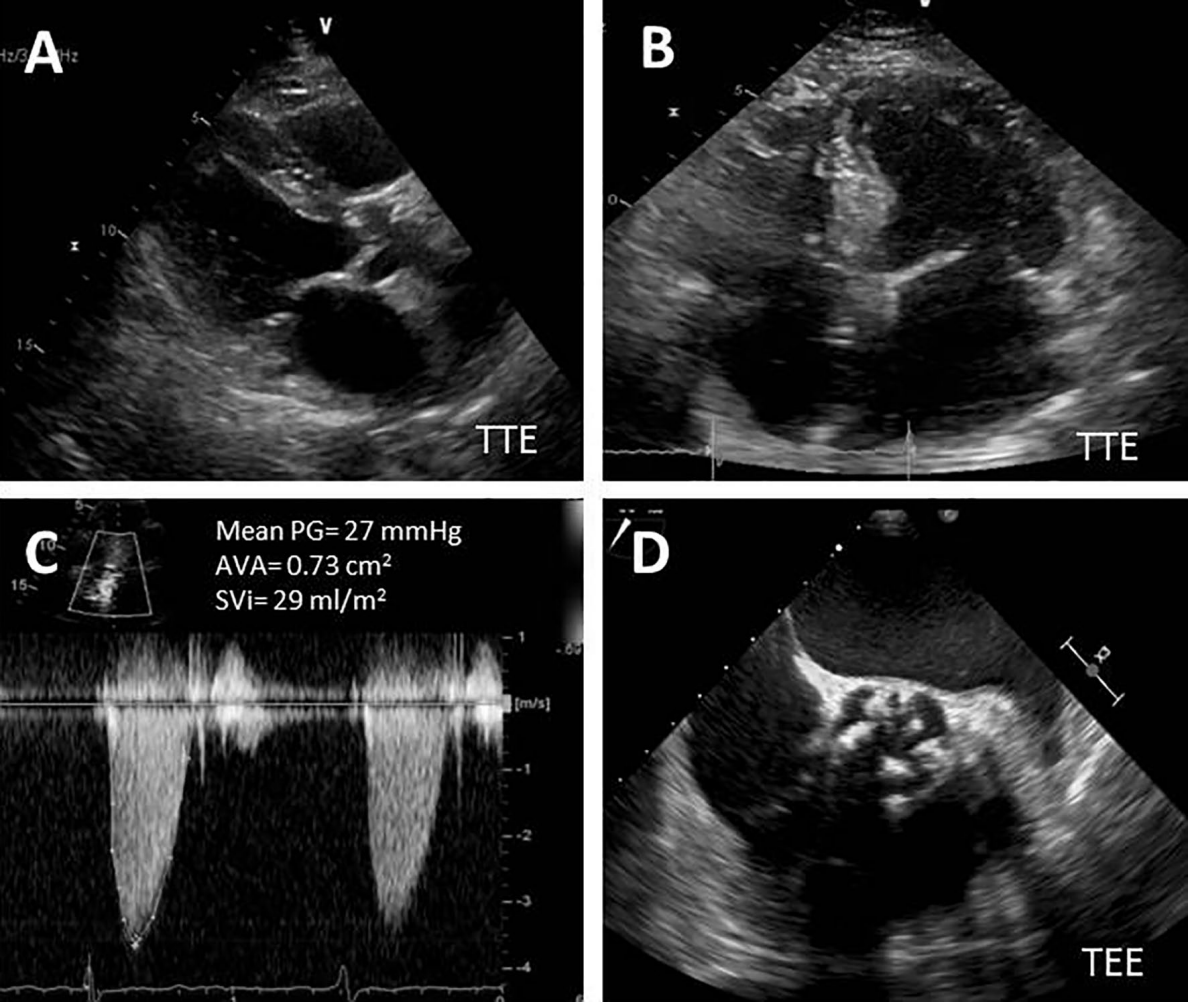
Illustration of echocardiographic image of a typical low-flow low-gradient severe aortic stenosis in clinical practice. **A** TTE shows left ventricular hypertrophy and restricted aortic valve opening; **B** TTE shows a small left ventricular size with dilated left atrium; **C** Doppler study shows a low-flow (SVi = 29 ml/m^2^), low-gradient (mean gradient = 27 mmHg) and a small calculated AVA = 0.73 cm^2^; **D** TEE often demonstrates calcified aortic valve

The American College of Cardiology/American Heart Association guidelines established the criteria for diagnosis of severe aortic stenosis (SAS) as a peak aortic peak velocity (V_max_) ≥ 4.0 m/s, a mean transaortic pressure gradient (MPG) ≥ 40 mmHg and an aortic valve area (AVA) ≤ 1.0 cm^2^ [1]. However, clinicians often encounter patients with discordant findings, such as a small AVA ≤ 1.0 cm^2^,  V_max_ < 4.0 m/s, and a MPG < 40 mmHg [2, 3]. When patients with discordant findings have preserved left ventricular ejection fraction (LVEF), they are often referred as low-gradient severe aortic stenosis (LG SAS) and preserved LVEF (Fig. 1). Some prior studies suggested that LG SAS may represent advanced disease and carry a poor prognosis with medical management alone, but other investigators found that clinical outcome of LG SAS with preserved LVEF parallels moderate AS [4–7]. Our group observed that LG SAS had overall better outcome than the gradient-AVA matched SAS (high gradient severe AS: V_max_ ≥ 4.0 m/s, MPG ≥ 40 mmHg, AVA ≤ 1.0 cm^2^), but worse than gradient-AVA matched moderate AS (true moderate AS: V_max_ < 4.0 m/s, MPG < 40 mmHg, AVA > 1.0 cm^2^) [8]. Other studies reported that LG SAS is associated with greater risk of mortality than high-gradient severe AS, and aortic valve intervention (AVI) might be beneficial in this subset of patients [3, 9–11]. However, the subgroup analysis of the PARTNER trial (Placement of Aortic Transcatheter Valves) failed to demonstrate a significant reduction in mortality in LG SAS up to 2 years [12]. In two more recent studies, one showed that AVI improved LV global longitudinal strain [13], but the other did not show significant improvement in LV global longitudinal strain, LV mass or neurohormonal activation [14]. With such a discrepancy in natural history, the choice of treatment of LG SAS needs further investigation [15]. Our prior study showed echocardiographic and clinical characteristics may play a role in prediction of prognosis in low-gradient AS [16]. Therefore, we hypothesized that clinical outcome in patients with LG SAS and preserved LVEF may be associated with multiple risk factors, comorbidities and certain echocardiographic features. Prior study has established that the Society of Thoracic Surgeons (STS) score was a powerful tool for predicting long-term outcome and for selecting patients with asymptomatic severe AS for aortic valve surgery [17]. EuroSCORE II study showed that combination of multiple risk factors could reliably predict mortality in patients who undergo cardiac surgery [18]. However, it is unknown if EuroSCORE II would be useful for predicting outcome in patients with low-gradient AS and preserved LVEF, who underwent AVI or conservative management respectively. The aim of the study was to assess whether combination of echocardiographic assessment and EuroSCORE II could be useful for stratifying patients with LG SAS and preserved LVEF.

## Methods

### Patients

The study included consecutive patients (age ≥ 18 years) with LG SAS (AVA ≤ 1.0 cm^2^, MPG < 40 mmHg V_max_ < 4.0 m/s) and preserved LVEF (≥ 50%) who underwent echocardiography at Wake Forest Baptist Medical Center from January 2011 to November 2016. Exclusion criteria included severe aortic regurgitation, severe mitral regurgitation, bicuspid aortic valve, or previous aortic valve surgeries. The study was approved by the Institutional Review Board.

Clinical data, including symptoms and comorbidities were obtained from electronic medical record reviews. Renal dysfunction was defined by eGFR < 60 ml/min. AVI was defined as either surgical or transcatheter aortic valve replacement. All-cause mortality was identified through cross-referencing the Social Security Death Index. According to the 2017 ESC/EACTS guidelines, the risk stratification of patients with aortic stenosis can be classified into a high-risk or low-risk group with the cutoff value of 4% EuroSCORE II [19]. The current study used the online EuroSCORE II calculation for all patients (http://www.euroscore.org).

### Echocardiography

Comprehensive 2-dimensional and Doppler transthoracic echocardiograms were performed according to the American Society of Echocardiography guidelines. LV volume, LVEF, LV mass and relative wall thickness were calculated from 2D echocardiography measurements. The aortic valve V_max_ and MPG were measured using continuous-wave Doppler. The left ventricular outflow tract (LVOT) diameters were measured at the aortic valve annulus in the parasternal long-axis view at mid-systole. LVOT velocity–time integral (VTI) was obtained in the apical 3 or 5-chamber view with pulsed-wave Doppler. AVA calculations were performed based on Doppler measurements. SV index (SVi) is defined as SV divided by body surface area.

### Statistical analysis

Continuous variables were reported as mean ± SD and compared with Student’s test. Categorical variables were reported as a percentage and compared using the chi-square test. The primary end point of the present study was all-cause mortality. Kaplan–Meier analysis with log-rank testing was used to compare cumulative survival between each group. A Cox proportional hazards multivariable model with forward stepwise regression was used to determine the independent predictors of all-cause mortality in the whole study group and the variables with a p value < 0.1 in univariate analysis were incorporated into the multivariate model. Statistical analysis was performed using IBM SPSS software V. 22.0.

## Results

In the current study, a total of 323 consecutive patients with LG SAS (AVA ≤ 1.0 cm^2^, MPG < 40 mmHg, V_max_ < 4.0 m/s) and preserved LVEF (≥ 50%) were identified in our echocardiography database. Patients were divided into two groups according to surgical risks stratification by EuroSCORE II: a high-risk group (EuroSCORE II ≥ 4%, n = 115) and a low-risk group (EuroSCORE II < 4%, n = 208).

Baseline clinical characteristics in each group are listed in Table 1. Mean EuroSCORE II was 2.2 ± 0.9% in the low-risk group and 7.9 ± 5.2% in the high-risk group (p < 0.001). The majority of patients in both groups had low flow state (SVi: 32.1 ± 9.6 m/m^2^ vs. 32.9 ± 9.4 ml/m^2^, p > 0.05).The patients in the high-risk group were older and more symptomatic than those in the low-risk group. Compared with the low-risk group, the high-risk group had a higher prevalence of heart failure, atrial fibrillation, renal dysfunction, coronary artery disease, peripheral vascular disease, previous transient ischemic attack/stroke, larger left atrium (LA) and higher pulmonary systolic artery pressure (sPAP). There were no significant differences in LV mass index, LVEF, mean pressure gradient, AVAi and stroke volume index (SVi) between the high-risk and the low-risk group (Table 2).

**Table 1:** Clinical characteristic of patients with EuroSCORE II ≥ 4% or EuroSCORE II < 4%

	EuroSCORE II < 4% (n = 208)	EuroSCORE II ≥ 4%(n = 115)	*P* value
Age (years)	74 ± 8	82 ± 7	< 0.001
Gender (male)	91 (43.8%)	40 (34.8%)	0.116
Body surface area (m^2^)	1.86 ± 0.25	1.77 ± 0.23	0.003
Symptoms			
Symptomatic	98 (47.1%)	85 (73.9%)	< 0.001
Dyspnea	65 (31.3%)	63 (54.8%)	< 0.001
Angina	17 (8.2%)	9 (7.8%)	0.913
Syncope	9 (4.3%)	8 (6.9%)	0.311
Comorbidities			
Atrial fibrillation	66 (31.7%)	59 (51.3%)	0.001
Hypertension	166 (79.8%)	101 (87.8%)	0.068
Diabetes mellitus	80 (38.5%)	44 (38.3%)	0.972
Dyslipidemia	110 (52.9%)	59 (51.3%)	0.785
Heart failure	82 (39.4%)	93 (80.9%)	< 0.001
Previous coronary artery disease	66 (31.7%)	65 (56.5%)	< 0.001
Previous percutaneous coronary intervention	26 (12.5%)	26 (22.6%)	0.018
Previous coronary artery bypass grafting	1 (0.5%)	33 (28.7%)	< 0.001
Chronic obstructive pulmonary disease	47 (22.6%)	25 (21.7%)	0.859
Previous transient ischemic attack/stroke	35 (16.8%)	30 (26.1%)	0.047
Renal dysfunction	79 (37.9%)	79 (68.7%)	< 0.001
Peripheral vascular disease	10 (4.8%)	19 (16.5%)	< 0.001
EuroSCORE II	2.2 ± 0.9%	7.9 ± 5.2%	< 0.001

**Table 2:** Hemodynamic and echocardiographic parameters of patients with EuroSCORE II ≥ 4% or EuroSCORE II < 4%

Parameters	EuroSCORE II < 4% (n = 208)	EuroSCORE II ≥ 4% (n = 115)	P value
Systolic blood pressure (mmHg)	139 ± 23	137 ± 28	0.357
Diastolic blood pressure (mmHg)	69 ± 12	66 ± 14	0.039
AVAi (cm^2^/m^2^)	0.47 ± 0.09	0.46 ± 0.10	0.694
Peak (mmHg)	42 ± 18	44 ± 17	0.335
MPG (mmHg)	22.5 ± 9.7	23.3 ± 9.5	0.463
LVOT diameter at annulus (mm)	18.6 ± 2.5	18.6 ± 2.4	0.916
SVi (mL/m^2^)	32.1 ± 9.6	32.9 ± 9.4	0.449
LV end-diastolic diameter (mm)	43.0 ± 5.9	41.7 ± 6.6	0.074
LV end-systolic diameter (mm)	28.9 ± 5.6	28.2 ± 6.6	0.310
Ejection fraction (%)	58.1 ± 4.6	58.2 ± 4.3	0.849
LV mass (g)	194 ± 63	187 ± 62	0.321
LV mass index (g/m^2^)	104 ± 31	105 ± 30	0.743
Relative wall thickness	0.57 ± 0.14	0.61 ± 0.16	0.024
Left atrial diameter (mm)	40.6 ± 8.1	43.9 ± 8.0	< 0.001
Pulmonary artery systolic pressure (mmHg)	37.8 ± 18.3	47.1 ± 19.5	< 0.001

**Fig. 2:**
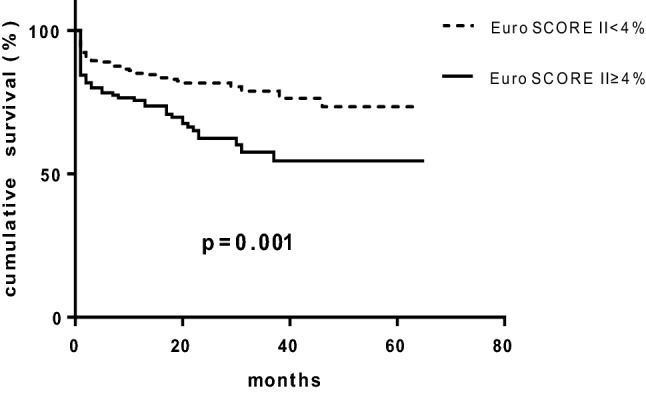
Kaplan–Meier curves analysis of overall survival in EuroSCORE II < 4% and EuroSCORE II ≥ 4% in the whole study group

**Fig. 3:**
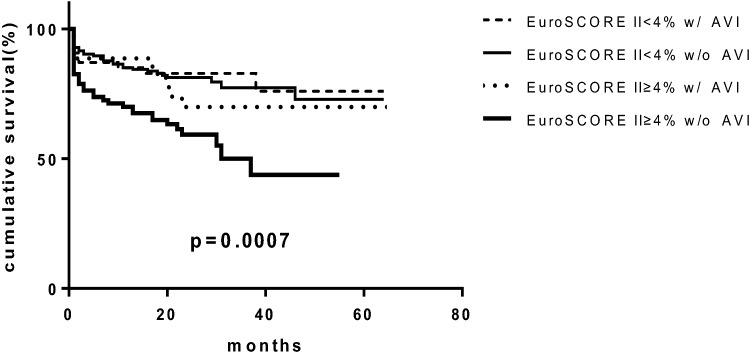
Kaplan–Meier curves in the both low-risk and high-risk patients with or without AVI. High-risk patients without AVI had the lowest survival (*w/AVI* with aortic valve intervention; *w/o AVI* without aortic valve intervention)

Mean follow-up duration was 2 ± 1.3 years. During the follow-up period, a total of 89 patients (27.5%) had AVI (60 surgical valve replacements and 29 transcatheter aortic valve replacements). Among them, there were no significant differences in AVI between the low-risk patients (n = 54, 25.9%) the high-risk patients (n = 35, 30.4%, p = 0.389). Death occurred in 85 patients (26%) during follow-up, of whom 42 (20.2%) were in the low-risk group, 43 (37.4%) were in the high-risk group (p = 0.001). Kaplan–Meier analysis showed that overall 2-year cumulative survival was significantly lower in the high-risk group than in the low-risk group. (62.3% vs. 81.7%, p = 0.001, Fig. 2). The subgroup analysis showed that without AVI, 2-year all-cause mortality was significantly higher in the high-risk group than low-risk group (81.2% vs. 59.2% p < 0.001) and the 2-year cumulative survival tended to be higher in the high-risk group with AVI than those without AVI (70% vs. 59% p = 0.065, Fig. 3). However, the 2-year cumulative survivals were similar between the low-risk patients with (82.8%) or without AVI (81.2%, p = 0.68, Fig. 3). The univariate analysis showed that age, heart failure, COPD, left atrial size, SVi and systolic pulmonary artery pressure were associated with all-cause mortality. However, traditional echocardiographic measures for the severity of AS (peak pressure gradient, mean gradient and AVA) were not associated with all-cause mortality. The multivariate analysis showed that heart failure, renal dysfunction and SVi were independently associated with increased risk for all-cause mortality (Table 3).

**Table 3:** Univariate and multivariate analyses for predictors of all-cause mortality in all patients with LG SAS and preserved LVEF

Variable	Univariable analysis		Multivariable analysis	
	HR (95% CI)	*p* value	HR (95% CI)	*p* value
Age	1.03 (1.0–1.05)	0.048		0.637
Male	1.35 (0.88–2.06)	0.171		N/A
Atrial fibrillation	1.41 (0.92–2.16)	0.115		N/A
Heart failure	2.84 (1.73–4.65)	< 0.001	2.62 (1.59–4.31)	< 0.001
COPD	1.90 (1.20–2.99)	0.006		0.066
Renal dysfunction	2.07 (1.32–3.23)	0.001	1.82 (1.16–2.87)	0.009
Left atrial diameter	1.04 (1.01–1.07)	0.004		0.053
Ejection fraction	1.01 (0.96–1.06)	0.791		N/A
SVi	0.98 (0.96–1.0)	0.056	0.98 (0.95–0.99)	0.033
sPAP	1.01 (1.0–1.02)	0.008		0.164
AVAi	0.92 (0.08–10.27)	0.949		N/A
MPG	0.99 (0.97–1.02)	0.521		N/A

*HR* hazard ratio, *95% CI* 95% confidence interval, *COPD* chronic obstructive pulmonary disease

## Discussion

The current study evaluated the utility of echocardiographic findings and EuroSCORE II in the risk stratification of patients with LG SAS and preserved LVEF. Based on these analyses, several salient points emerged: First, patients with a high EuroSCORE II experienced higher all-cause mortality than patients with low EuroSCORE II (Fig. 2); Second, AVI tended to improve survival in the high-risk group; Third, The low-risk patients (lower EuroSCORE II) had relatively high 2-year cumulative survival regardless of AVI.

Debate continues regarding the most appropriate management of patients with LG SAS and preserved LVEF [15]. AVI may not make the patients with low-flow LG SAS feel better or survive longer according to some investigators’ opinion [15]. Some studies reported that LG SAS with preserved LVEF represents a relatively benign form of AS with outcomes similar to moderate AS. Jander et al. [5] in the Simvastatin and Ezetimibe in Aortic Stenosis study compared LG SAS patients with moderate AS and found LG SAS patients with preserved LVEF had similar outcomes in terms of aortic valve events, cardiovascular events, and cardiovascular death. In a separate study, the outcome of low-flow, low-gradient SAS was shown to be similar to moderate AS but better than high gradient severe AS [4]. However, these studies mainly included relatively low-risk patient population [20] and excluded those with significant cardiovascular risk factors and comorbidities, such as coronary heart disease, heart failure, diabetes mellitus, atrial fibrillation, and renal insufficiency. In a real-world practice, patients with LG SAS and preserved LVEF represent a heterogeneous population and often exhibit clustering of clinical characteristics [21–25]. We also observed that these patients often share many common high-risk features including elderly, high prevalence of hypertension, coronary artery disease, atrial fibrillation, dyspnea, dilated left atrium, pulmonary hypertension and heart failure (Table 1). Therefore, it may be reasonable to speculate that patients with LG SAS and high EuroSCORE II may have occult LV dysfunction, decreased cardiac reserve and/or afterload intolerance, which may be associated with unfavorable clinical outcome, particularly in the presence of significant AS.

Echocardiography plays an important role in diagnosing LG SAS with preserved LVEF. Many studies attempted to identify echocardiographic parameters that would predict outcome of LG SAS with preserved LVEF. AVAi, pressure gradients, dobutamine testing and other parameters have been analyzed, but the results were not always consistent [26–30]. The current study also showed that the traditional echocardiographic measures for the severity of AS (AVAi and pressure gradients) were not significant in predicting all-cause mortality in LG SAS with preserved LVEF. However, SVi calculated by echocardiography was independently associated with mortality, which was consistent with a recent study [31]. Pathophysiological connection between SVi and mortality in LG SAS with preserved LVEF could be multifactorial [32]. Decrease in SVi may lead to lower cardiac output, reduction in intrinsic LV systolic function, myocardial performance and myocardial global longitudinal strain which were associated with worse operative and long-term outcomes [33].

In this study with 2 years of clinical follow-up, AVI did not reduce all-cause mortality in low-risk patients, which was consistent with previous studies [5, 6]. For patients with low-gradient low-flow severe AS, AVI may not always improve quality of life or survival by expert’s opinions [15]. A recent study showed that AVA impacts prognosis only in those with high-gradient AS and preserved LVEF and whether AVA truly adds prognostic information over V_max_ or MPG is not clear in patients with LG SAS [34]. Although different theories and explanations have been proposed, the inherent variability in calculating SV by echocardiography may have a significant impact on the accuracy in diagnosing true SAS and/or classifying flow state in some patients [16, 35]. With the limitations of echocardiography in determining the true severity of AS based on AVA and in the absence of a high pressure gradient, conservative management of LG SAS patients with low EuroSCORE II may be considered as an option in a medium-term with close follow-up. This conservative approach allows further investigation of the severity of AS and the verification of SVi calculation for AS classification, which are important for the selection of appropriate management strategies [36]. However, AVI may be considered in those with higher EuroSCORE II in LG SAS for whom the lack of a high pressure gradient may suggest a potentially compromised left ventricle.

### Study limitations

Similar to most of LG SAS studies, the current study has its own limitations. The study is retrospective and non-randomized. Decisions of performing AVI were made by individual cardiothoracic surgeons and/or interventional cardiologist. Due to the lack of reliable information on the cause of death, this study is unable to identify the specific cause or factors related to terminal events (cardiac or non-cardiac death). Therefore, all-cause mortality was used as an endpoint in this study. Limitations of echocardiography in diagnosis of LG SAS include potential underestimation of stroke volume and AVA. These could potentially lead to misclassification of LG SAS patients [35, 36]. However, the technical limitations are not specific to either the low-risk or high-risk groups. It also remains possible that some clinical or echocardiographic predictors may become statistically significant with increasing patient numbers or in a different patient population with longer follow-up.

## Conclusions

In patients with LG SAS and preserved LVEF, a higher EuroSCORE II predicted worse prognosis with conservative management and AVI tended to improve survival. However, in those with a low EuroSCORE II, AVI did not show significant improvement in survival in comparison with conservative management during 2-year follow-up. SVi independently predicted survival, while AVA and mean gradient did not. Further study is needed to determine the long-term benefit of AVI in the low-risk patients with LG SAS and preserved LVEF.
